# Identifying phenotypes in interstitial lung disease using group-based trajectory modelling

**DOI:** 10.5588/ijtld.22.0417

**Published:** 2023-04-01

**Authors:** S. Takata, S. Komukai, T. Hoshino, H. Tabuchi, K. Masuhiro, M. Yaga, Y. Shirai, Y. Mitsui, Y. Abe, T. Kuge, K. Fukushima, H. Kida, A. Kumanogoh

**Affiliations:** 1 Department of Respiratory Medicine and Clinical Immunology, Osaka University Graduate School of Medicine, Osaka, Japan; 2 Department of Integrated Medicine Biomedical Statistics, Osaka University Graduate School of Medicine, Osaka, Japan; 3 Clinical Laboratory, Kakogawa Central City Hospital, Kakogawa, Japan; 4 Laboratory for Clinical Investigation, Osaka University Hospital, Osaka, Japan; 5 Department of Respiratory Medicine, National Hospital Organization Osaka Toneyama Medical Center, Osaka, Japan; 6 Department of Immunopathology, World Premier International Research Center Initiative, Immunology Frontier Research Center, Osaka University, Osaka, Japan; 7 Integrated Frontier Research for Medical Science Division, Institute for Open and Transdisciplinary Research Initiatives, Osaka University, Osaka, Japan; 8 Center for Infectious Disease for Education and Research, Osaka University, Osaka, Japan; 9 Japan Agency for Medical Research and Development – Core Research for Evolutional Science and Technology (AMED–CREST), Osaka University, Osaka, Japan; 10 Center for Advanced Modalities and DDS (CAMaD), Osaka University, Osaka, Japan

Dear Editor,

Group-based trajectory modelling (GBTM) analysis is a semi-parametric mixture model for longitudinal data used to identify latent groups. This analytical method has been used to discover new phenotypes in heterogeneous diseases.^1^ In GBTM, we first assume the number of latent trajectories exist in the cohort and the order of polynomial function of time trajectories these are expressed. Next, the parameters of the function are calculated, based on the maximum likelihood for the whole dataset without distinguishing individual patients. The probability for each patient of belonging to each trajectory is then estimated. Using this method, different time courses of pulmonary function have been identified in chronic obstructive pulmonary disease and amyotrophic lateral sclerosis.^2^,^3^ Interstitial lung disease (ILD) is a heterogeneous condition caused by several distinct diseases, including idiopathic pulmonary fibrosis (IPF), connective tissue disease-associated ILD (CTD-ILD), interstitial pneumonia with autoimmune features, etc. Today, ILD is classified into progressive fibrosing ILD (PF-ILD) and non-PF-ILD, according to criteria in a large clinical trial of nintedanib.^4^ However, the number of latent trajectories of the percentage of predicted normal forced vital capacity (%FVC) in heterogeneous ILD remains unknown. In this study, we performed GBTM analysis to objectively find latent groups (including the PF phenotype) among patients with chronic ILD using serial %FVC data. We also examined whether those groups would reflect their survival outcomes.

We first retrospectively selected 530 patients with chronic ILD who had three or more measurements of %FVC^5^ within 5 years of their first visit to Osaka University Hospital, Osaka, Japan, from September 1993 to August 2018. Patients were excluded if they had lung transplantation (*n* = 8), lung resection (*n* = 21), no high-resolution computed tomography (HRCT) data (*n* = 13), little information in the medical records (*n* = 25) or pulmonary malignancy at the first pulmonary function test without lung resection (*n* = 1). The remaining 462 patients were included in the GBTM analysis. A follow-up period of 5 years after the first %FVC assessment was chosen to reduce the amount of missing data, and the median number of time points during 5 years in the development cohort was 3 (interquartile range [IQR] 3–5). The effect of patients dropping out for certain reasons, such as death, was dealt with by applying this extended GBTM (hereafter referred to as eGBTM).^6^

For eGBTM, we tried all permutations of two-group to six-group linear, quadratic, cubic and quartic trajectory models. The first pulmonary function test was the starting point. The times between the starting point and subsequent pulmonary function tests were approximated to multiples of 3 months. The best model was determined based on the balance of model fit and clinical usefulness. We selected the six-group linear trajectory model. The trajectories were as follows: very high/stable (*n* = 7, 1.5%): intercept 127.23 (95% confidence interval [CI] 120.95 to 133.51), slope 0.059 (95% CI−0.17 to −0.29); high/stable (*n* = 61, 13.2%): intercept 105.63 (95% CI 102.49 to 108.76), slope 0.007 (95% CI −0.075 to 0.088); normal/slow decline (*n* = 178, 38.5%): intercept 89.71 (95% CI 87.84 to 91.58), slope −0.087 (95% CI −0.14 to −0.033); low/slow decline (*n* = 123 26.6%): intercept 74.78 (95% CI 72.82 to 76.75), slope −0.11 (95% CI −0.17 to −0.046); low/rapid decline (*n* = 54, 11.7%): intercept 66.21 (95% CI 62.89 to 69.53), slope −0.28 (95% CI −0.38 to −0.18); and very low/stable (*n* = 39, 8.4%): intercept 46.82 (95% CI 42.41 to 51.22), slope −0.11 (95% CI −0.16 to −0.05) (Figure 1A). We performed Kaplan–Meier analysis to examine the prognosis of patients belonging to each trajectory, and showed that the patients in the low/rapid decline trajectory had the worst overall survival rate among all trajectories (Figure 1B).

**Figure:**
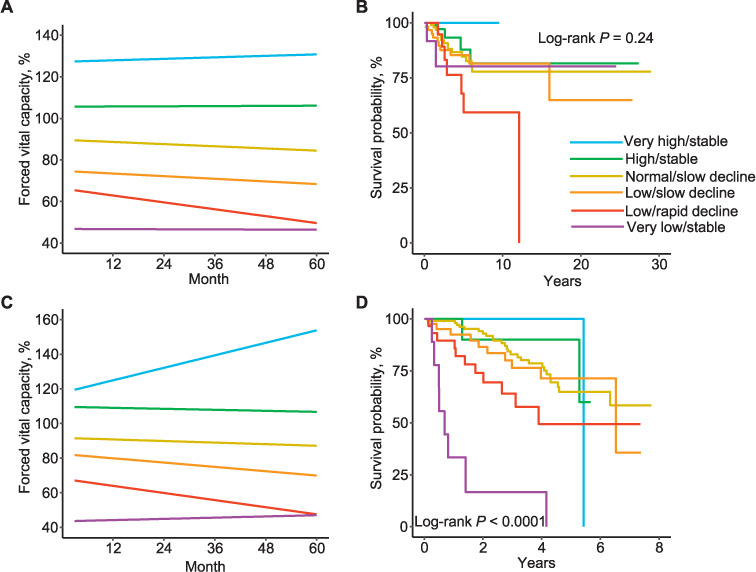
Trajectories of the percentage of predicted normal forced vital capacity (%FVC) and overall survival in the development cohort. A) All patients with chronic interstitial lung disease (n = 462) in the development cohort were classified on the basis of their trajectory of %FVC by group-based trajectory model. The following six trajectories were found: very high/stable (n = 7), high/stable (n = 61), normal/slow decline (n = 178), low/slow decline (n = 123), low/rapid decline (n = 54) and very low/stable (n = 39). B) Overall survival of each trajectory in the development cohort. C) Trajectories of %FVC of the six-trajectory linear model in the validation cohort: very high/stable (n = 10), high/stable (n = 22), normal/slow decline (n = 63), low/slow decline (n = 64), low/rapid decline (n = 42), and very low/stable (n = 20). D) Overall survival of each trajectory in the validation cohort.

To validate our method of subclassifying patients with ILD by the time course of %FVC, data from a second patient population were collected from the National Hospital Organization Osaka Toneyama Medical Center, Osaka, Japan. A total of 284 patients with chronic ILD who had at least three %FVC measurements within 5 years from January 2012 to July 2019 were initially included. We excluded patients who had lung resection (*n* = 47), no HRCT data (*n* = 5) or pulmonary malignancy at baseline without lung resection (*n* = 11). The remaining 221 patients were included in the second eGBTM analysis. The median number of time points during 5 years in the development cohort was 4 (IQR 3–6). The median age of patients in this cohort was 71 years (IQR 64.0–76.0); this was significantly higher than that of the first cohort (median 66 years, IQR 57.0–72.0) (*P* < 0.001). The proportion of IPF was significantly higher (24.0% vs. 33.3%, *P* = 0.017) and the proportion of CTD-ILD significantly lower (37.7% vs. 18.6%, *P* < 0.001) in this second cohort. Despite such demographic differences, eGBTM with the six-trajectory linear model showed similar trajectories in the second cohort. The trajectories were as follows: very high/stable (*n* = 10, 4.5%): intercept 117.78 (95% CI 111.28 to 124.28), slope 0.60 (95% CI 0.36 to 0.84); high/stable (*n* = 22, 10.0%): intercept 109.69 (95% CI 105.64 to 113.74), slope −0.049 (95% CI −0.18 to 0.077); normal/slow decline (*n* = 63, 28.5%): intercept 91.70 (95% CI 88.85 to 94.56), slope −0.076 (95% CI −0.17 to −0.02); low/slow decline (*n* = 64, 29.0%): intercept 82.37 (95% CI 79.18 to 85.55), slope −0.21 (95% CI −0.30 to −0.12); low/rapid decline (*n* = 42, 19.0%): intercept 68.11 (95% CI 64.48 to 71.74), slope −0.34 (95% CI−0.48 to −0.21); and very low/stable (*n*=20, 9.0%): intercept 43.53 (95% CI 38.18 to 48.88), slope 0.058 (95% CI −0.21 to 0.33) (Figure 1C). Similar to the first cohort, these trajectories were also significantly associated with the prognosis (*P* < 0.001) (Figure 1D).

This study has a few limitations. Our eGBTM analysis was not adjusted for pharmacological interventions. In the Kaplan–Meier analysis, the analysed group was limited to the patients who survived for at least the period for determining the group.

In daily clinical practice, we recently divided patients with chronic ILD into two groups, PF-ILD and non-PF-ILD. However, in this study, we have shown that the six-group model is a better fit. The two-group models are likely to be too simple because chronic ILD consists of several distinct diseases, diagnosed at different stages. Furthermore, even when a chronic ILD patient has a moderate restrictive lung function, prognosis is not uniform. Moreover, even when a patient with chronic ILD has normal %FVC we must observe the patient carefully, taking into account the prognosis after 5 years. The next step is to predict which trajectory the patient will follow.
